# Enhancing the Physicochemical, Thermal, and Technological Properties of Freeze-Dried Welsh Onion Leaf Juice: Influence of Maltodextrin and Gum Arabic as Carrier Agents

**DOI:** 10.3390/polym17060801

**Published:** 2025-03-18

**Authors:** Carolina Medina-Jaramillo, Alex López-Córdoba

**Affiliations:** Grupo de Investigación en Bioeconomía y Sostenibilidad Agroalimentaria, Escuela de Administración de Empresas Agropecuarias, Facultad Seccional Duitama, Universidad Pedagógica y Tecnológica de Colombia, Carrera 18 con Calle 22, Duitama 150461, Colombia; carolina.medina02@uptc.edu.co

**Keywords:** *Allium fistulosum* L., bioactive compounds, food ingredients, freeze drying, vegetable juice

## Abstract

Fresh Welsh onions are widely used in food formulations due to their distinctive flavor and biological properties, but their high perishability limits their industrial applications. In this study, powdered Welsh onion leaf juices were obtained through freeze-drying, with and without maltodextrin (MD) and gum arabic (GA) as carrier agents. MD was chosen for its high solubility and neutral taste, while GA was selected for its ability to improve powder stability and dispersibility. Powders were obtained using a completely randomized design to evaluate the effects of five MD:GA ratios (0:100, 25:75, 50:50, 75:25, and 100:0) on their physicochemical and technological properties. The addition of carriers enabled the formation of fine, homogeneous powders with higher water solubility. All formulations exhibited low water activity (<0.4) and moisture content (<7%). Polyphenol content ranged from 2.60 to 3.53 mg GAE/g of dry matter, with a high recovery percentage (94–96%). DPPH^•^ scavenging activity was about 0.55 mg GAE/g of dry matter for all powders with carrier agents. Fourier-transform infrared (FTIR) analysis confirmed the presence of characteristic bands from both the carrier agents and the onion leaf juice, while thermogravimetric analysis (TGA) revealed enhanced thermal stability with carrier agents. Flowability tests showed that MD and MD:GA blends significantly improved powder handling.

## 1. Introduction

The increasing interest in the development of natural ingredients has been driven by a growing demand across various industrial sectors, including food, pharmaceuticals, and cosmetics [1,2]. This trend is particularly pronounced in the food industry due to consumer preferences for healthier options and a desire to avoid synthetic additives. Consequently, the exploration of plant-based sources for bioactive compounds has gained considerable traction, reflecting a broader movement towards sustainable and health-promoting food products [2].

Among the various plant species, *Allium fistulosum* L., commonly known as Welsh onion or spring onion, has emerged as a valuable candidate due to its widespread cultivation, bioactive compounds content, and biological properties [3]. However, the high moisture content of fresh Welsh onions makes them highly perishable, limiting their usability and storage stability [1,4].

Welsh onions consist of four main parts: root, stem, pseudostem, and leaves, with the leaves comprising up to 52% of fresh weight [5]. Previous research has shown that the leaves of Welsh onion are rich in nutrients, including carbohydrates, proteins, vitamins, and minerals, as well as bioactive compounds such as flavonoids, saponins, steroids, alliin, allicin, 4-hydroxybenzoic, and p-coumaric acid [6,7]. Some of these compounds have been associated with various health benefits, including antioxidant, antimicrobial, anti-inflammatory, and hypocholesterolemic properties, making them of particular interest for functional food applications [3,6,7].

Processing fresh vegetables into juice is a useful strategy to enhance their usability, as vegetable juices are commonly used as marinating agents and ingredients in soups, sauces, and salads [5,8]. Mechanical extraction is a widely used method for obtaining juice, offering several industrial and commercial advantages, including high extraction yields, cost-effectiveness, and adaptability to both large- and small-scale production [9,10]. However, research on Welsh onion juice remains limited.

Despite their potential applications, vegetable liquid juices are unstable and prone to degradation, requiring additional processing to facilitate its incorporation into food formulations. In this context, converting liquid juice into a powdered form through dehydration presents a promising strategy. This approach improves handling and extends stability, making it more suitable for industrial applications [2].

Freeze-drying is one of the dehydration technologies commonly used to produce powdered ingredients with favorable technological properties and extended shelf lives [11]. This process involves the removal of water through freezing and subsequent sublimation of ice under reduced pressure, which helps retain the integrity of bioactive compounds [12,13]. Compared to drying techniques such as spray-drying or oven-drying, freeze-drying offers superior retention of volatile compounds, color, and flavor, which are critical for functional food applications [11]. Furthermore, it results in highly porous powders with excellent rehydration properties, making them more suitable for use in the food industry [1,11,13,14].

However, freeze-dried plant-based powders often suffer from high hygroscopicity and stickiness, posing challenges in their handling and storage [15]. To address these issues, carrier agents are commonly used to improve the physical and technological properties of powders [15]. Maltodextrin, a polysaccharide obtained by the partial enzymatic or acid hydrolysis of starch, is one of the most used carrier agents for the freeze drying of food ingredients due to its high solubility, neutral taste, and ability to enhance powder flowability [15]. Gum arabic, a natural hydrocolloid, is known for its film-forming capacity and stabilizing properties, contributing to improved powder stability and dispersibility [14,16,17,18]. The combination of MD and GA has been demonstrated to synergistically enhance handling characteristics [14,16,17,18]. Although these carriers are widely used as coadjutants in food dehydration processes, their impacts on the freeze-drying of Welsh onion leaf juice remain unexplored. Therefore, this study aims to evaluate the effects of maltodextrin (MD) and gum arabic (GA) on the physicochemical, thermal, and technological properties of freeze-dried Welsh onion juices. The novelty of the current work lies in its focus on Welsh onion leaves, a previously underexplored resource, and the application of freeze-drying techniques combined with carrier agents to enhance the quality and usability of the resulting powders.

## 2. Materials and Methods

### 2.1. Raw Material and Reagents

Welsh onions (*Allium fistulosum* L.) were sourced from a local market (Aquitania, Boyacá, Colombia). Gum arabic (GA) and maltodextrin (MD) with a dextrose equivalent (DE) of 18–22 were obtained from Tecnas S.A. (Medellín, Colombia). Other reagents, such as Folin–Ciocalteu reagent, ethanol, and gallic acid, were sourced from PanReac (Barcelona, Spain) and Merck (Darmstadt, Germany), while 2,2-diphenyl-1-picrylhydrazyl (DPPH^•^) reagent came from Sigma-Aldrich (St. Louis, MO, USA). All chemicals used were of analytical grade.

### 2.2. Juice Extraction

Juice was extracted as described in a previous study [19]. Fresh Welsh onion leaves were ground using an electric grinder. The resulting material was then filtered to separate the juice, which was subsequently centrifuged, and vacuum filtered. The pH of the juice was measured using a digital pH meter (Oakton Instruments, Vernon Hills, IL, USA), while the soluble solids content (°Brix) was determined using an Atago PR 101 refractometer (Atago Co., Tokyo, Japan).

### 2.3. Preparation of Dispersions

Mixtures of Welsh onion leaf juice and carrier agents were prepared at an 80:20 ratio (weight-to-weight), with the MD:GA proportions varying as follows: 0:100, 25:75, 50:50, 75:25, and 100:0. These ratios were chosen based on previous reports in the literature [20] and our prior studies on freeze-dried juices [19,21].

The carrier materials were dissolved in the juice under constant stirring using an Ultra Turrax T25 homogenizer (IKA^®^ WERKE, Staufen, Germany).

### 2.4. Freeze Drying

The homogenized formulations were frozen at −20 °C for 24 h and freeze-dried at −55 °C under 0.1 mbar for 48 h using a Lyovapor L-200 freeze-dryer (BUCHI, Flawil, Switzerland). The dried cakes were ground into powders with a coffee grinder (Cuisinart, Stamford, CT, USA), sieved through a 60-mesh sieve, and stored in airtight containers.

### 2.5. Color Analysis

CIELAB parameters (L*, a*, b*) were measured with a Minolta colorimeter (Konica-Minolta CR-10, Osaka, Japan). Hue angles and chroma values were calculated using the recommended CIELAB equations.

### 2.6. Scanning Electron Microscopy

The morphological features of the freeze-dried Welsh onion leaf juice were analyzed using a ZEISS EVO MA10 microscope (Carl Zeiss SMT Ltd., Cambridge, UK). The samples were mounted on stubs, coated with a thin layer of gold, and examined at an acceleration voltage of 20 kV [22].

### 2.7. Fourier-Transform Infrared Spectroscopy (FTIR)

FTIR analysis was carried out using a JASCO FT/IR-4100 spectrometer (Hachioji, Tokyo, Japan) equipped with a diamond ATR module. Spectra were collected in the 4000–4500 cm^−1^ range at a resolution of 4 cm^−1^, with 24 scans per spectrum.

### 2.8. Total Polyphenol Content and DPPH^•^ Scavenging Activity

Total polyphenol content and DPPH^•^ scavenging activity were measured following the methodologies outlined in previous studies [7,21]. Polyphenol content was determined using the Folin–Ciocalteu method, with results expressed as gallic acid equivalents (GAE) per gram of dry solids. The recovery percentage of polyphenols was calculated as the ratio between the total phenolic compound concentration (dry basis) in the freeze-dried powders and that in the initial compound fed into the freeze-dryer.

Antioxidant activity was assessed using DPPH^•^ radicals according to the method described by Brand-Williams [23]. Liquid juice was appropriately diluted in distilled water, while powders were reconstituted in the same solvent. A 100 μL aliquot of each sample was then mixed with 3.9 mL of DPPH ethanol solution (25 mg DPPH/L). Absorbance was determined at 515 nm until the reaction reached a plateau. The results were expressed as mg GAE per gram of dry solids.

### 2.9. Thermogravimetric Analysis

TGA was conducted in an SDT-Q600 thermal analyzer (TA Instruments, New Castle, DE, USA) under a dry N_2_ atmosphere, heating the samples from 50 to 600 °C at a rate of 10 °C min^−1^.

### 2.10. Moisture Content, Water Activity, and Water Solubility

Moisture content was measured by drying samples at 105 °C to constant weight, while water activity was determined using an AquaLab Series 3 TE instrument (Pullman, WA, USA). Solubility was evaluated by mixing 1 g of freeze-dried juice with 100 mL of water under continuous stirring at 1000 rpm for 5 min (IKA RT5 magnetic stirrer, Staufen, Germany). The mixture was then centrifuged at 1500 rpm for 5 min, and the supernatant was dried at 105 °C until at a constant weight. Solubility was calculated as a percentage by dividing the dry weight by the initial sample mass.

### 2.11. Flow Properties

The flow features of the freeze-dried Welsh onion leaf juice were evaluated as reported in previous studies [24,25]. Loose bulk density was measured by allowing a known mass of powder (3–5 g) to flow freely into a 10 mL graduated cylinder and dividing the mass by the resulting volume. Tapped bulk density was determined by measuring the weight of the powder and its volume after tapping the cylinder until the volume stabilized [24].

The Hausner ratio and compressibility index were calculated using the procedures outlined by López-Córdoba et al. [24].

### 2.12. Statistical Analysis

A completely randomized design was followed with one factor (the MD:GA ratio) at five levels (0:100, 25:75, 50:50, 75:25, and 100:0). The response variables include physicochemical properties (e.g., color parameters, moisture content, solubility, and antioxidant activity) [26]. Statistical analysis was performed with Minitab v.16. (State College, PA, USA). Analysis of variance (ANOVA) and Tukey’s tests were applied with a 95% confidence level. All experiments were conducted in triplicate, and data were reported as mean ± standard deviation.

## 3. Results and Discussion

### 3.1. Juice Properties

Table 1 shows the physicochemical properties of the Welsh onion leaf juice. The juice exhibited a soluble solids content of 3.46 ± 0.06 °Brix and a dry solids percentage of 3.45%. Additionally, the juice displayed high water activity and pH values, characteristic of foods susceptible to microbial spoilage [27].

Regarding the CIELAB color attributes, the values obtained were consistent with its intense fern green coloration (Table 1). This intense green hue is likely attributed to the presence of chlorophyll and other phytochemicals, which contribute to the overall visual appeal of the juice [28].

The total polyphenol content and the DPPH^•^ scavenging activity of Welsh onion leaf juice were similar to those reported for various onion genotypes [29], but higher than those reported for other vegetable juice such as red beetroot juice [30]. This finding highlights the potential of Welsh onion leaf juice as a rich source of bioactive compounds, particularly polyphenols, which are known for their antioxidant properties and associated health benefits [3].

### 3.2. Appearance and Color Attributes of the Freeze-Dried Welsh Onion Leaf Juice

**Figure 1:**
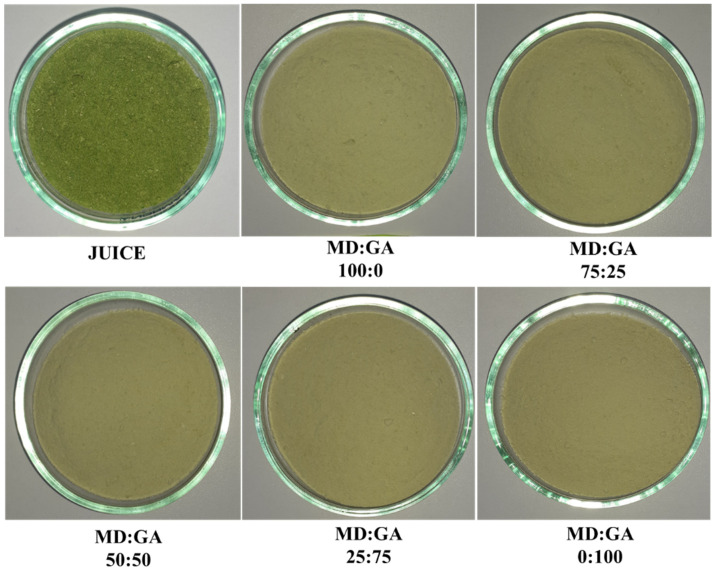
Images of the freeze-dried Welsh onion leaf juice samples obtained without and with the addition of carrier agents. MD: maltodextrin; GA: gum arabic.

Figure 1 shows the appearance of the freeze-dried Welsh onion leaf juice obtained without and with the addition of carrier agents. Visual appearance showed differences in color, brightness, and texture across the samples. Freeze-dried Welsh onion leaf juice samples without carrier agents showed darker hues, reduced brightness, and coarser, more irregular appearance. In contrast, powders formulated with carrier agents displayed finer, more homogeneous appearances (Figure 1).

The results from the CIELAB color analysis agreed with the visual observations (Table 2). The powders formulated with MD alone and an MD:GA ratio of 75:25 showed higher lightness (L* values) compared to the freeze-dried Welsh onion leaf juice powders without carrier agents, indicating a shift toward brighter and less saturated colors. These results suggest that MD contributes more effectively to maintaining a lighter powder, likely due to its inherent whiteness [31]. Similarly, Tkacz et al. reported an increase in L* values after incorporating maltodextrin into sea buckthorn juice powders [14]. In contrast, no significant differences were observed in lightness values between the juices and the powders produced with GA alone, as well as those with MD:GA ratios of 50:50 and 25:75.

Furthermore, the incorporation of carrier agents in all samples resulted in an increase in the a* coordinate values, indicating a shift toward a less intense green color, while the b* coordinate values decreased, suggesting a shift towards a less saturated yellow.

Freeze-dried powders had significantly lower chromas (*p* < 0.05) than the freeze-dried juice powders without carrier agents, suggesting more muted color profiles in the final products. The 100% MD powder exhibited higher chroma (21.26 ± 2.06) than 100% GA powder (16.43 ± 1.14), suggesting that MD helped retain more color intensity. Meanwhile, powders formulated with a combination of carrier agents displayed chroma values that fell between those of the powders made with individual carriers (Table 2). These findings underscore the significant impact of carrier agents on the color attributes of freeze-dried Welsh onion leaf juice, which may influence consumer perception and product quality.

### 3.3. Morphological Properties of the Freeze-Dried WELSH Onion Leaf Juice

**Figure 2:**
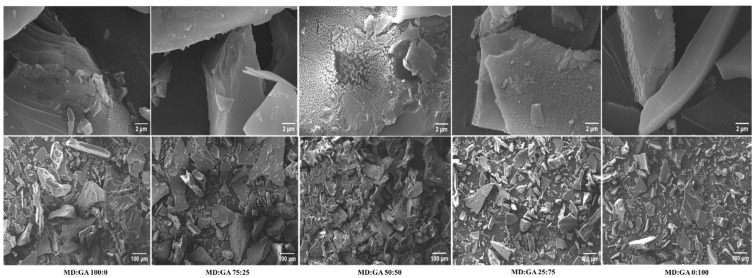
Images of scanning electron microscopy (SEM) of freeze-dried Welsh onion leaf juice powders obtained with different ratios of carrier agents (maltodextrin (MD) and gum arabic (GA)).

Figure 2 shows SEM images of the freeze-dried Welsh onion leaf juice powders obtained with different ratios of carrier agents. All samples exhibited the characteristic broken-glass morphology, featuring large particles of irregular shapes, which is typical of freeze-dried products [14,21]. According to Hay et al., this irregular, flake-like morphology of freeze-dried powders often provides a larger surface area, and this could contribute to improved dispersibility in liquid matrices, a critical factor when considering food formulations [14].

The observed variations in particle size can likely be attributed to the grinding process employed post-freeze-drying. This finding aligns with previous studies that have noted how grinding techniques can influence the particle size distribution and morphology of freeze-dried products [21].

### 3.4. FTIR Spectra Analysis

**Figure 3:**
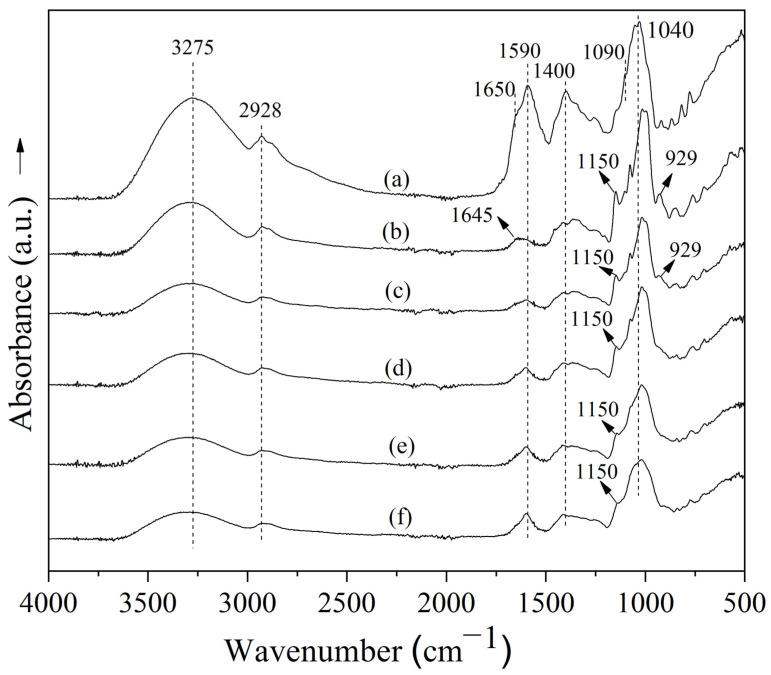
FTIR spectra of freeze-dried Welsh onion leaf juice obtained with and without carrier agents (maltodextrin (MD) and gum arabic (GA)): (a) Onion leaf juice without carrier agents, (b) MD:GA 100:0, (c) MD:GA 75:25, (d) MD:GA 50:50, (e) MD:GA 25:75, and (f) MD:GA 0:100.

FTIR spectra of the freeze-dried Welsh onion leaf juice powders, both with and without carrier agents, are shown in Figure 3. The IR spectrum of the Welsh onion leaf juice without carrier agents showed an absorption band at 3275 cm^−1^ associated with O–H stretching vibrations (Figure 3). Moreover, the spectrum exhibits a band at 2928 cm^−1^, associated with the stretching and bending vibrations of C–H bonds (Figure 3, a). Peaks located between 1400 cm^−1^ and 1300 cm^−1^ suggest the presence of carbonyl group vibrations [7]. Other significant bands were found at 1650 cm^−1^and 1590 cm^−1^, which corresponds to the C=O stretching vibrations of carbonyl groups, likely attributed to various compounds such as flavonoids and other phenolic constituents found in onion juice (Figure 3). Furthermore, signals at 1040 cm^−1^ and 1090 cm^−1^ could be associated with alcohol functional groups [7].

All freeze-dried Welsh onion leaf juice samples with carrier agents showed characteristic bands of their wall materials (MD and GA) and of Welsh onion leaf juice (Figure 3) [21], suggesting that the addition of carrier agents did not affect the chemical composition of the Welsh onion leaf juice. Maltodextrin has been identified as exhibiting specific absorption bands at 3300 cm^−1^ (O–H stretching), 2905 cm^−1^ (asymmetric C–H_2_ stretching), 1641 cm^−1^ (indicating free carboxyl groups), 1150 cm^−1^ (C–O stretching), 1005 cm^−1^ (C–O stretching), and 929 cm^−1^ (C–O–C stretching associated with glycosidic bonds and CH_2_ out-of-plane bending) [21]. These spectral features are comparable to those observed in gum arabic, which displays bands at 3429 cm^−1^ (O–H stretching), 2930 cm^−1^ (a low-intensity peak linked to free carboxyl groups), 1590 cm^−1^ (C=O stretching), and 1039 cm^−1^ (C–O stretching) [21].

### 3.5. Polyphenol Content and DPPH^•^ Scavenging Activity of the Freeze-Dried Welsh Onion Leaf Juice

Table 3 shows the total polyphenol contents of freeze-dried Welsh onion leaf juice obtained with and without carrier agents. The freeze-dried juice without carrier agents showed the highest total polyphenol content (19.45 ± 0.56 mg GAE g^−1^), which was expected since no carrier agents were present to dilute the phenolic compounds. This value is lower than those reported by Goyal et al. for onion leaf powders obtained through vacuum-drying and grinding (32.79–36.37 mg GAE/g), but higher than those reported by Aquino et al. for freeze-dried spring onion leaf extract of the “*Cipollotto Nocerino*” variety (0.76–1.43 mg GAE/g dry weight) [5,32]. These differences can be attributed to variations in cultivar type and juice processing methods.

The recovery percentage of polyphenols in the freeze-dried juice was approximately 94%, compared to the liquid juice, suggesting that the freeze-drying process did not cause a significant reduction in the content of these bioactive compounds (Table 3).

For freeze-dried juice with carrier agents, the total polyphenol content ranged from 2.60 to 3.53 mg GAE/g of dry matter. In all samples the average polyphenol recovery percentage was 96% (*p* > 0.05), indicating that neither the presence of carrier agents nor the freeze-drying process led to significant polyphenol losses.

On the other hand, the highest total polyphenol content was observed in samples with an MD:GA ratio of 0:100 and 25:75, while the lowest values among the powders were found in the 100:0 formulation (Table 3). This suggests that GA plays a more protective role in preserving phenolic compounds during the freeze-drying process compared to MD. Meanwhile, powders formulated with an MD:GA ratio of 75:25 and 50:50 exhibited intermediate polyphenol contents between those of powders made with single carriers (Table 3). This finding aligns with the results reported by Laureanti et al., which indicated that the addition of GA to the formulation enhanced the concentration of phenolic compounds [11]. The increase in total polyphenol content (TPC) observed in freeze-dried juice powders with gum arabic may be attributed to its molecular structure. This biopolymer consists of a highly branched sugar-based structure with a minor proportion of protein covalently attached to the carbohydrate backbone, which enhances its ability to form protective films and thus retain Welsh onion polyphenols more effectively [33,34]. Furthermore, its emulsifying characteristics improve the solubility and dispersion of polyphenols, minimizing their loss during the freeze-drying process [14].

Despite the variations in total polyphenol content, no significant differences were observed in DPPH^•^ scavenging activity among the samples (Table 3). This behavior could be attributed to the slight increase in polyphenol content, which may not have been sufficient to significantly alter the antioxidant properties measured by the DPPH^•^ assay. The presence of less effective phenolic compounds could also contribute to this phenomenon, indicating that not all phenolic compounds contribute equally to antioxidant activity.

### 3.6. Thermogravimetric Analysis of the Freeze-Dried Welsh Onion Leaf Juice

**Figure 4:**
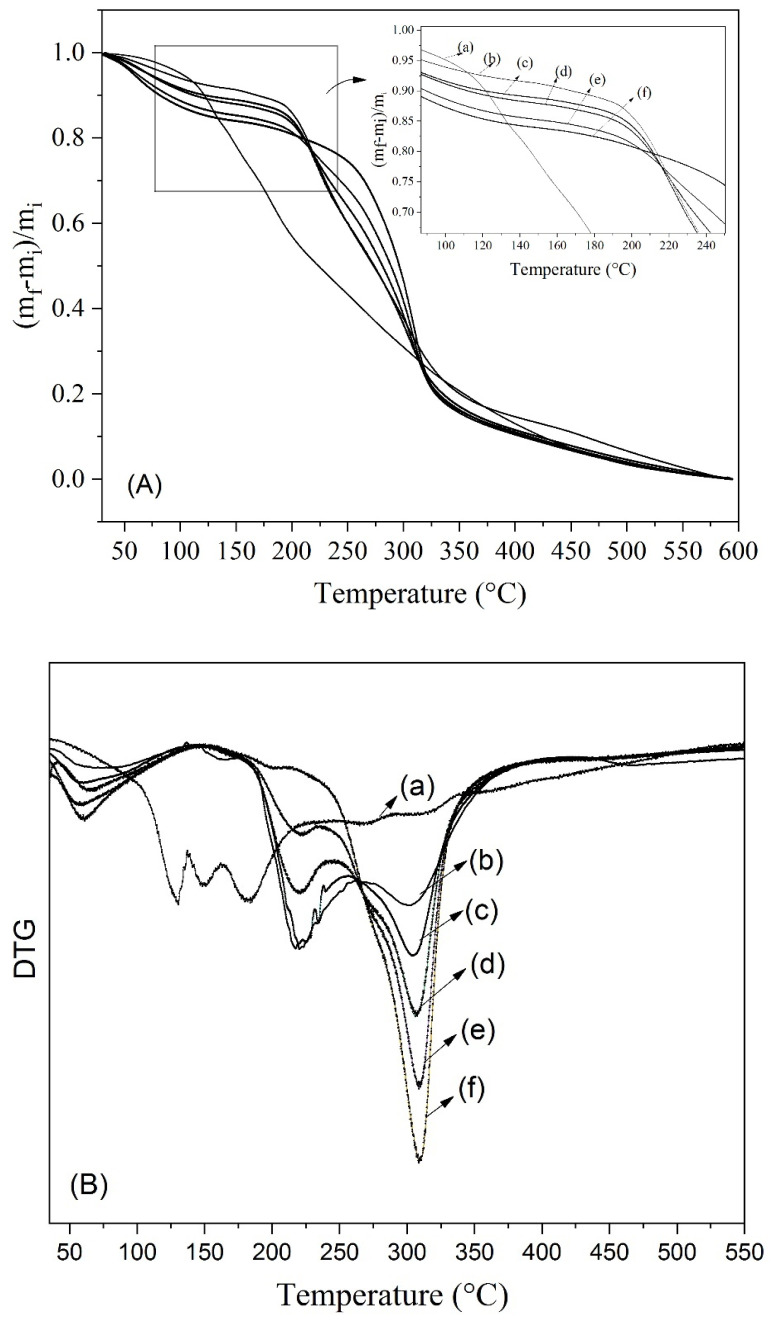
Thermogravimetric analysis (**A**) and DTG curves (**B**) of freeze-dried Welsh onion leaf juice obtained with and without carrier agents (maltodextrin (MD) and gum arabic (GA)): (a) Onion leaf juice without carrier agents, (b) MD:GA 100:0, (c) MD:GA 75:25, (d) MD:GA 50:50, (e) MD:GA 25:75, and (f) MD:GA 0:100.

The thermogravimetric analysis (TGA) provided insights into the thermal behavior of the freeze-dried Welsh onion leaf juice (Figure 4). All samples exhibited a small amount of weight loss between 50 °C and 150 °C, which was attributed to water loss [11]. Furthermore, the freeze-dried Welsh onion leaf juice without carrier agents exhibited rapid thermal decomposition starting at temperatures above 138 °C. This decomposition is likely due to the breakdown of bioactive constituents present in the juices, such as flavonoids and phenolic compounds, which are known to be sensitive to heat [11,35].

The freeze-dried juice samples produced with carrier agents demonstrated a significant delay in the decomposition of their components, with the onset of thermal decomposition above 180 °C. The samples produced with maltodextrin alone exhibited thermal events between 180 °C and 267 °C, as well as between 267 °C and 400 °C (Figure 4). In contrast, the samples obtained with gum arabic alone displayed a single thermal event occurring between 213 °C and 385 °C (Figure 4). The presence of gum arabic in the blend with maltodextrin was particularly noteworthy, as it resulted in a reduction in weight loss between 180 °C and 267 °C, indicating an enhancement in the thermal stability of the Welsh onion leaf juice. Similar findings were reported by Tang et al. who characterized powdered gardenia yellow pigment using various encapsulating agents, including maltodextrin, gum arabic, isolated soy protein, and whey protein isolate [35]. Their study observed a displacement of endothermic peaks to higher temperatures, which was attributed to the protective effects of the wall materials during heating.

### 3.7. Moisture Content, Water Activity, and Water Solubility

The moisture content of the freeze-dried Welsh onion leaf juice powders ranged from 4.94% to 6.55%. MD:GA 75:25 ratio exhibited the lowest moisture content, showing a significant reduction compared to the juice without carriers and the formulations with MD:GA ratios of 50:50 and 0:100 (Table 4). This suggests that a moderate addition of GA may lead to powders with lower moisture content, contributing to enhancing their handling and storage stability [27,36]. However, increasing GA content beyond a 75:25 ratio did not lead to further reductions in moisture, as seen in the 50/50, 25/75, and 0/100 MD/GA formulations, which had moisture levels comparable to the juice alone. Conversely, the formulation with MD alone had a moisture content like the juice without carriers, indicating that MD alone does not significantly decrease the moisture content.

The water activity (a_w_) values of the freeze-dried Welsh onion juice powders ranged from 0.27 to 0.37, with no significant differences among the formulations (*p* > 0.05) (Table 4). This indicates that the incorporation of different maltodextrin (MD) and gum arabic (GA) ratios did not substantially affect the final a_w_ of the powders. All samples exhibited a_w_ values below 0.4, which is desirable for ensuring microbiological stability and prolonging shelf life by minimizing enzymatic and oxidative degradation [27,36].

The onion leaf juice powders without carriers exhibited solubility of approximately 72%, whereas in all powders containing maltodextrin and/or gum arabic, this parameter increased to approximately 88% (Table 4). This enhancement in solubility can be attributed to the inherent properties of the carrier agents, which possess high water solubility themselves [37]. The increased solubility is advantageous for applications where quick rehydration is desired, such as in instant food products or dietary supplements. The results of this study are consistent with previous research indicating that the incorporation of maltodextrin and gum arabic as carrier agents significantly enhances the solubility of freeze-dried powders [19,21].

### 3.8. Flowability Analysis

Table 5 shows the flow properties of freeze-dried Welsh onion leaf juice obtained with and without carrier agents.

The loose-bulk density and tapped-bulk density of the freeze-dried Welsh onion leaf juice powders ranged from 385.93 to 413.94 kg/m^3^ and from 452.82 to 474.52 kg/m^3^, respectively, with no significant differences among the freeze-dried Welsh onion leaf juice powders obtained with and without carrier agents (*p* > 0.05). These results indicate that the addition of maltodextrin (MD) and gum arabic (GA) in different ratios did not substantially alter the powder’s packing properties [38,39]. The slight variations observed suggest that the carrier agents did not induce major structural modifications in particle arrangement or cohesiveness [38,39].

Powders with a Hausner ratio between 1.00 and 1.11, and a Carr index below 10% are classified as having excellent flowability [40]. Values within the ranges of 1.12–1.18 for the Hausner ratio, and 11–15% for the Carr index, indicate good flowability [40]. In contrast, powders with Hausner ratios of 1.19–1.25, or Carr indexes of 16–20% exhibit fair flowability [40].

The flow properties of the freeze-dried Welsh onion leaf juice powders, evaluated through their Hausner ratios and Carr indexes, varied depending on the MD:GA ratio. The juice powder without carrier agents exhibited a Hausner ratio of 1.22 and a Carr index of 18.26%, classifying it within the “fair” flowability range. This suggests that the powder tended to form cohesive particles, which could hinder flowability.

Formulations containing MD and GA displayed improved flow properties. The MD:GA ratios of 100:0, 75:25, 50:50, and 25:75 exhibited Hausner ratio and Carr index values within the “good” flowability range. In contrast, the sample with a 0:100 MD:GA ratio showed a Hausner ratio of 1.20 and a Carr index of 16.98%, indicating fair flowability, similar to the juice without carrier agents. This suggests that GA alone does not significantly improve powder flow properties compared to MD or MD–GA combinations.

Overall, incorporating MD or MD–GA blends markedly enhances a powder’s flowability, making it more suitable for handling and processing. This improvement can be attributed to the ability of these agents to reduce particle cohesion and enhance the overall powder structure, facilitating easier manipulation. These findings align with previous studies on the impact of carrier agents on the flow properties of freeze-dried powder [11].

## 4. Conclusions

Welsh onion leaf juice was successfully transformed into fine, homogeneous, and easily manageable powders through freeze-drying with maltodextrin (MD) and/or gum arabic (GA). Among the formulations, MD was more effective than GA in preserving a lighter powder color and maintaining color intensity. Polyphenol recovery was approximately 94% in freeze-dried juice without carrier agents and averaged 96% in powders with carriers, indicating minimal polyphenol loss due to freeze-drying. However, the incorporation of carrier agents diluted the polyphenol content in the final powders. The highest total phenolic content was observed in samples with an MD:GA ratio of 0:100 and 25:75, whereas the MD-only formulation had the lowest. Despite these variations, antioxidant activity (DPPH• scavenging) remained statistically similar across all samples.

The addition of carrier agents significantly improved thermal stability. Juice without carriers decomposed rapidly above 138 °C, whereas formulations containing GA and MD–GA blends exhibited delayed decomposition, enhancing overall stability. Moisture content was lowest in the MD:GA 75:25 ratio, though increasing GA beyond this proportion did not further reduce moisture. Water activity values (0.27–0.37) remained below 0.4 across all formulations, ensuring microbial stability. Solubility improved from approximately 72% in powders without carriers to around 88% in formulations containing MD and/or GA. Additionally, the incorporation of carrier agents enhanced flow properties, with MD and MD–GA blends significantly improving powder flowability, making these formulations more suitable for handling and processing.

## Figures and Tables

**Table 1:** Characteristics of the Welsh onion leaf juice.

Physicochemical Properties	Value
Soluble solids content (°Brix)	3.46 ± 0.06
Dry solid content (%)	3.45 ± 0.04
Water activity	1.01 ± 0.05
pH	5.80 ± 0.01
Color coordinates (CIELAB)	L* = 41.95 ± 1.70
	a* = −24.22 ± 3.89
	b* = 47.10 ± 0.29
	h = 117.11 ± 3.58
	c = 53.04 ± 2.04
Total polyphenol content (mg GAE/g dw)	20.73 ± 0.90
DPPH^•^ scavenging activity (mg GAE/g dw)	2.16 ± 0.05

**Table 2:** Color attributes of freeze-dried Welsh onion leaf juice powders obtained with and without carrier agents.

MD/GA Concentration (%)	L*	a*	b*	*h*	Chroma	ΔE
Juice *	52.35 ± 0.07 ^c^	−13.95 ± 0.49 ^a^	39.90 ± 2.68 ^a^	109.28 ± 0.56 ^a^	42.26 ± 2.64 ^a^	-
100/0	60.01 ± 1.38 ^a^	−5.54 ± 2.75 ^b^	17.45 ± 3.36 ^b^	106.78 ± 5.93 ^a^	21.26 ± 2.06 ^b^	25.26 ± 3.86 ^a^
75/25	59.05 ± 1.62 ^a,b^	−4.47 ± 1.81 ^b^	17.13 ± 2.73 ^b^	104.36 ± 4.59 ^a^	19.63 ± 1.58 ^b,c^	25.64 ± 2.83 ^a^
50/50	57.11 ± 2.34 ^c,a,b^	−3.68 ± 1.59 ^b^	18.41 ± 1.51 ^b^	101.28 ± 4.82 ^a^	18.83 ± 1.55 ^b,c^	24.43 ± 1.46 ^a^
25/75	56.92 ± 2.54 ^c,a,b^	−4.24 ± 2.17 ^b^	17.64 ± 1.46 ^b^	103.24 ± 6.23 ^a^	18.23 ± 1.71 ^b,c^	24.88 ± 1.79 ^a^
0/100	55.90 ± 2.68 ^c,b^	−4.18 ± 1.71 ^b^	17.9 ± 2.95 ^b^	102.75 ± 3.89 ^a^	16.43 ± 1.14 ^c^	24.45 ± 3.37 ^a^

* Freeze-dried Welsh onion leaf juice without carrier agents; MD: maltodextrin; GA: gum arabic. Different letters within the same column indicate significant differences (*p* < 0.05).

**Table 3:** Total polyphenol content, polyphenol recovery percentage, and DPPH^•^ scavenging activity of freeze-dried Welsh onion leaf juice obtained with and without carrier agents.

MD/GA Concentration (%)	Total Phenolic Content (mg GAE g^−1^)	Total Polyphenol Recovery (%)	DPPH Radical Scavenging Capacity (mg GAE g^−1^)
Juice *	19.45 ± 0.56 ^a^	93.7 ± 1.3 ^a^	3.28 ± 0.12 ^a^
100/0	2.60 ± 0.11 ^e^	93.7 ± 3.5 ^a^	0.55 ± 0.14 ^b^
75/25	2.92 ± 0.06 ^d^	95.3 ± 9.5 ^a^	0.56 ± 0.08 ^b^
50/50	3.20 ± 0.21 ^c,d^	95.3 ± 9.1 ^a^	0.50 ± 0.13 ^b^
25/75	3.27 ± 0.11 ^b,c^	98.0 ± 5.9 ^a^	0.50 ± 0.09 ^b^
0/100	3.53 ± 0.17 ^b^	99.1 ± 7.1 ^a^	0.54 ± 0.08 ^b^

* Freeze-dried Welsh onion leaf juice without carrier agents; MD: Maltodextrin; GA: gum arabic. Different letters within the same column indicate significant differences (*p* < 0.05).

**Table 4:** Moisture content, water activity, and water solubility of freeze-dried Welsh onion leaf juice obtained with and without carrier agents.

MD/GA Concentration (%)	Moisture Content (%)	Water Activity (a_w_)	Water Solubility (%)
Juice *	6.55 ± 0.07 ^a^	0.27 ± 0.01 ^a^	72.42 ± 1.85 ^b^
100/0	5.63 ± 0.49 ^a,b^	0.35 ± 0.06 ^a^	88.68 ± 0.78 ^a^
75/25	4.94 ± 0.32 ^b^	0.30 ± 0.09 ^a^	88.62 ± 0.55 ^a^
50/50	6.50 ± 0.63 ^a^	0.37 ± 0.01 ^a^	87.78 ± 0.64 ^a^
25/75	5.84 ± 0.56 ^a,b^	0.33 ± 0.02 ^a^	87.37 ± 0.34 ^a^
0/100	6.36 ± 0.51 ^a^	0.30 ± 0.02 ^a^	87.55 ± 0.82 ^a^

* Freeze-dried Welsh onion leaf juice without carrier agents; MD: maltodextrin; GA: gum arabic. Different letters within the same column indicate significant differences (*p* < 0.05).

**Table 5:** Flow properties of freeze-dried Welsh onion leaf juices obtained with and without carrier agents (maltodextrin (MD) and gum arabic (GA)).

MD:GA Concentration (%)	Loose-Bulk Density (kg m^−3^)	Tapped-Bulk Density (kg m^−3^)	Hausner Ratio	Carr Index (%)
Juice *	385.93 ± 0.12 ^a^	472.26 ± 7.71 ^a^	1.22 ± 0.02 ^a^	18.26 ± 1.36 ^a^
100/0	392.51 ± 13.89 ^a^	461.66 ± 15.75 ^a^	1.17 ± 0.02 ^a,b^	14.96 ± 1.78 ^a,b^
75/25	396.36 ± 15.53 ^a^	467.32 ± 22.36 ^a^	1.18 ± 0.02 ^a,b^	15.14 ± 1.66 ^a,b^
50/50	413. 94 ± 13.21 ^a^	474.36 ± 17.08 ^a^	1.14 ± 0.01 ^b^	12.72 ± 0.85 ^b^
25/75	390.50 ± 30.82 ^a^	452.82 ± 31.48 ^a^	1.16 ± 0.02 ^b^	13.80 ± 1.71 ^b^
0/100	393.83 ± 13.45 ^a^	474.52 ± 17.04 ^a^	1.20 ± 0.02 ^a^	16.98 ± 1.80 ^a^

* Freeze-dried Welsh onion leaf juice without carrier agents; MD: Maltodextrin; GA: gum arabic. Different letters within the same column indicate significant differences (*p* < 0.05).

## Data Availability

The original contributions presented in this study are included in the article. Further inquiries can be directed to the corresponding author.
